# Exploring Videoconferencing for Older Adults with Cognitive Concerns Using a Dramaturgical Lens

**DOI:** 10.1145/3663548.3675647

**Published:** 2024

**Authors:** Ruipu Hu, Ge Gao, Amanda Lazar

**Affiliations:** College of Information Studies, University of Maryland, United States; College of Information Studies, University of Maryland, United States; College of Information Studies, University of Maryland, United States

**Keywords:** Videoconferencing, Older Adults, Cognitive Concerns, Dementia, Design for Aging, Sociological Theory, Accessibility

## Abstract

While videoconferencing is a promising technology, it may present unique challenges and barriers for older adults with cognitive concerns. This paper presents a deconstructed view of videoconferencing technology use using a sociological dramaturgical framework developed by Erving Goffman. Our study recruited 17 older adults with varying cognitive concerns, employing technology discussion groups, interviews, and observations to gather data. Through a reflexive thematic analysis, we explore videoconferencing use among older adults with cognitive concerns, focusing on three major areas: the ”performances and roles” where users adapt to new roles through videoconferencing; the ”backstage,” which involves the physical and logistical setup; and the ”frontstage,” where people communicate through audio and visual channels to present a desired impression. Our discussion generates insights into how deconstructing these elements can inform more meaningful and accessible HCI design.

## 1 Introduction

Researchers have long been interested in supporting remote synchronous communication for older adults through videoconferencing [4, 12, 20, 38, 50, 59], a focus that accelerated during and after the pandemic [30, 36, 44, 63, 68]. Videoconferencing technology has been useful to provide psychosocial support, particularly to reduce isolation [53, 63], and provide access to healthcare [41] and physical training [30] services. For individuals with cognitive concerns, uses have also included remote diagnosis [24, 34, 62], therapeutic intervention [56], and online caregiver support groups [60].

While videoconferencing is seen as having great potential for older adults, challenges with this medium such as audio and inter-operability [33] have been noted for decades. Other examples of issues include management needed from users to compensate for poor sound and video [11, 47], and communication becoming complicated when videoconferencing blurs the lines between private and public spaces, leading to breaches of personal boundaries [21].

For older adults, these issues are likely compounded. In addition to differences in rates of technology use [19], attitudes towards older people as technologically incapable compose another barrier: where older people are not even always given the chance to participate in videoconferencing activities [64]. Concerns about access issues for older adults are so significant that Sin et al. attribute an increased digital divide to the transition to virtual-only offerings during the pandemic, including the transition from face-to-face interaction to videoconferencing [52].

Older people with cognitive concerns may be particularly vulnerable to the barriers described above. Past research has shown the ways family and professional caregivers must be deeply involved in facilitating remote communication for people with dementia [14, 28]. However, people can experience a broad spectrum of memory and cognitive concerns, ranging from self-perceptions of memory changes to mild, moderate, and severe cognitive concerns that arise due to causes that vary from depression to dementia [6, 26, 39, 69, 70]. When broadening our focus to older adults with a spectrum of cognitive concerns, it is less clear how individuals, who may not have formal caregivers, navigate videoconferencing.

One productive lens to study communication avenues such as videoconferencing has been through theatrical metaphors as put forward by Erving Goffman [22]. This dramaturgical framework was initially used to explain face-to-face communication, with parallels drawn between everyday interaction and theater performances where individuals carefully manage the impressions of their audience [22]. These insights have been extended to mediated interaction, with video and audio-mediated environments establishing new norms for people to organize interaction [48]. In the field of Human-computer interaction (HCI), researchers have applied Goffman’s framing to applications as diverse as online dating [18], social media use [9], and information and communication in the military setting [3]. Other work has studied how individuals present different identities, such as related to disability [18] and chronic pain [42]. When older adults utilize videoconferencing technologies, they may also engage in self-presentation, particularly as they cope with cognitive concerns. Our motivation for working with Goffman’s framing began with the aim to capture insights in our data not typically described in research, particularly around how cognitive concerns presented themselves and how they were managed. As such, we ask the following question with this study: *How can Goffman’s dramaturgical framework be applied to study videoconferencing technology use by older adults with cognitive concerns?*

To answer this question, we conducted two tech discussion groups, ten semi-structured interviews, and five observations. Participants all above the age of 65 with varying levels of cognitive concerns, which spanned from subjective cognitive decline to dementia. Adopting Goffman’s framing allowed us to deconstruct the data into three sets of theatrical metaphors. We found that videoconferencing served as a tool for sustaining, adapting, and embracing new “***roles***” amidst cognitive changes supporting diverse performances including familial, religious, and others. Participants engaged in critical “***backstage***” work selecting devices and using “*cues*” like emails and handwritten notes to prepare for frontstage performances. Finally, audio and video channels in the “***frontstage***” were main avenues for impression management.

Our paper offers the following contributions. First, we reveal how older adults with cognitive concerns are engaging in videoconferencing, a mediated communication technology. The discussion situates these findings in past research uncovering meaningful engagement through videoconferencing. Second, by adopting Goffman’s notions of frontstage and backstage, we expose the work involved in videoconferencing. This reveals the complexity of supporting more accessible videoconferencing and some ways forward for research. Finally, we examine how cognitive changes appear in videoconferencing through Goffman’s framing, with role changes and impression management as important domains. We found theatrical metaphors to be useful in shifting the narrative away from deficit models that often dominate the discourse around aging and technology [61] by recontextualizing technical issues and cognitive conditions.

## 2 Related Work

### 2.1 The potential of videoconferencing and associated accessibility needs

Videoconferencing allows people in different locations to hold face-to-face meetings without having to move to a single location together. Videoconferencing has been explored as a potential approach to mitigate social isolation and enhance social support among the general older adult population [4, 12, 20, 38, 50, 58, 59]. Especially amid the COVID-19 pandemic, videoconferencing emerged as a tool to bridge the physical disconnect imposed by necessary safety measures [30, 36, 40, 44, 53, 63, 68], providing more comfort for older adults to continue engaging in activities they participated in before the pandemic. For example, Lee et al. revealed that older adults experienced convenience and motivation using videoconferencing-delivered online exercise since the COVID-19 pandemic [30].

Many of the studies evaluating videoconferencing for older adults have focused on this technology’s potential to ameliorate social isolation [58, 59]. A systematic review of videoconferencing among populations in elderly care institutions found evidence regarding its feasibility [40]. The authors emphasize the effectiveness of videoconferencing in reducing social isolation [40]. Another body of work highlights the potential of videoconferencing in enhancing healthcare access for older adults, especially in care home settings. Newbould et al. suggest that videoconferencing can improve access to a range of services, encourage continuity of care, remove the inconvenience of travel, and provide better access for those with physical disabilities [41]. Within this body of work, Tsai et al. discovered that videoconferencing conversations notably enhanced emotional support by providing feelings of care, empathy, love, and trust [59].

Videoconferencing has been found to be promising for some older adults, yet there remain questions around its accessibility in a number of dimensions. Some research grounded in the context of remote work seeks to support accessibility in teams [1, 71]. Other work investigates how functionalities of videoconferencing systems present unique opportunities for improved accessibility [31, 32, 72]. Researchers have also focused on the support that is needed to make videoconferencing more accessible [15, 66]. While the above research has examined accessibility for different populations, more research is needed to study videoconferencing with older adults with cognitive concerns.

The literature exhibits an increasing interest in the potential of videoconferencing to support older adults with cognitive impairment, primarily to administer cognitive interventions that could help manage cognitive decline [24, 27, 34, 60, 62, 73], with some work providing assessment on their cognitive status and conditions [62]. Research has also utilized videoconferencing to support caregivers of people with dementia [22]. However, the study of the use of videoconferencing has been limited, especially for individuals experiencing more mild cognitive concerns [6, 26, 39, 69, 70].

### 2.2 Goffman’s theory in HCI and accessibility technologies

Erving Goffman’s dramaturgical theory [22] likens social interaction to a theatrical performance. In this framework, individuals are seen as performers who strategically present themselves to manage others’ impressions. As discussed by theorists Blumer [7] and Strauss [55], Goffman’s theory is often associated with symbolic interactionism, a sociological theory emphasizing that meaning is created through language, interaction, and subjective interpretation. Goffman’s framework has been useful in HCI to understand user behavior in technology environments [3, 9, 23, 35, 48, 74].

One of the core concepts in Goffman’s work involves the notions of “*frontstage*” and “*backstage*”. The frontstage represents “*where the performance is given*” (page 107, [22]). The backstage involve behind-the-scenes actions that are “*related to the performance but inconsistent with the appearance fostered by the performance*,” which are usually more private behaviors without the audience’s gaze (page 112 & 134, [22]). Some research has extended Goffman’s concept of stages to examine interactions facilitated by technology. For example, Ling explores how mobile phone calls establish “parallel frontstages [23],” while Cooper discusses merging public and private spaces [74]. In these scenarios, the boundary between personal and public interaction blurs, as private conversations often take place within public areas. Similarly, Aoki et al. has expanded on the idea of multiple frontstages within the setting of a combat information center. This work demonstrates how systems enabling multi-party communication can introduce complexities in team-work dynamics [3]. Impression management, another integral part of Goffman’s work describing how people self-present, was used in later research study emerging technology. Marwick and Boyd employ Goffman’s work to understand how users present themselves in online communities [35]. Rette’s work on mobile phone communication extends Goffman to study synchronous mediated interaction, arguing that a situational focus is less relevant to asynchronous media [48].

Some past work points to the potential of Goffman’s framework to investigate older adults’ identities. Brewer and Piper highlight Goffman’s work on self-presentation, discussing how older bloggers evolve their online identities through content creation and sharing. The authors suggest future work to support older adults’ identities online [9]. Our study picks up this call by focusing on role formation, adaptation, and the use of videoconferencing affordances to convey impressions.

## 3 Methods

Below, we describe our approach to data collection and analysis. All procedures were approved by our university’s Institutional Review Board (IRB).

### 3.1 Recruitment

Our data collection extended over a period of time that included caution associated with in-person interaction during the COVID-19 pandemic, as well as the period following. We recruited through collaborations with three entities: a residential community which included people living independently and in assisted living (Community A), a local community service center offering classes and activities tailored to older adults in a particular ethnic/racial group (Center B), and an online non-profit support group (Forum C) founded and governed by persons living with dementia.

Our engagement with Community A and Forum C occurred during the first data collection phase from October 2022 to May 2023. A personal contact supported the trust building necessary to recruit from assisted living. The collaboration with Center B took place in the second phase, from March to April 2024, after a more extended period required to establish trust and identify mutual research interests.

To recruit individuals for interviews and observations, we utilized word-of-mouth, personal contacts (through program managers or administrators at organizations), and flyers with study information. When potential participants reached out to us for further information about the study, we provided them information about the study via email or phone calls. To recruit discussion group participants, we included criteria in our posters encompassing any type of memory concern. These posters were distributed around Community A. To accommodate the preferred language in Center B, we translated the posters into Mandarin. Interviews and discussion groups with participants in Center B were also all conducted in Mandarin due to participant preference. Verbal consent was secured for participants in the Tech Discussion Group as required by the IRB. For all other sessions, consent forms were signed before any data collection began. Additionally, we arranged informal meetings for the participants in assisted living in Community A to allow them to become acquainted with the first author in advance of the first session, a method suggested by a staff member with close ties to the community.

### 3.2 Data Collection

We collected data through two discussion groups, ten interviews, and five observations. During tech discussion groups and interviews, we captured data through audio recordings and field notes. We gathered data from observation sessions through field notes, photos, and video recordings. Below, we provide more information about each approach to data collection:

#### Tech Discussion Group.

The discussion group took place in person at Community A and Center B. This session included two parts. The first part involved participants engaging in a discussion of their thoughts on interacting with videoconferencing technology. Subsequently, participants engaged in a group activity to discuss their everyday use application of videoconferencing, covering topics such as common usage and patterns, which led to a more in-depth group discussion. Tech discussion groups enabled us to gain broad insights regarding videoconferencing, particularly in its everyday application and common challenges. This was particularly useful to explain the uses of videoconferencing in section 4.1.

#### Interviews.

We conducted 1–1 interviews in different settings to accommodate participants’ preferences, including in their homes, in communal spaces, or remotely via Zoom. Interviews were semi-structured. Our primary objective was to delve into each participant’s personal experiences and perceptions of videoconferencing. Topics explored included their emotions, motivations, challenges faced, and specific needs related to videoconferencing. The in-depth semi-structured interviews provided a more personal and potentially safer space than discussion groups for participants to voice sensitive topics like concerns, personal experiences, and details about their cognitive conditions.

#### Observation.

Following the interview, participants were invited to partake in an observation session where they would demonstrate their use of videoconferencing technology while the researcher observed. This observation session was scheduled either immediately after the interview or at a separate time, taking into consideration the participants’ need for a cognitive break after the interview. This data collection method allows us to see participants’ videoconferencing from a different perspective, where we could become privy to “*backstage*” activities since we were no longer the main audience. A brief post-observation interview was conducted with participants who were willing to do so.

### 3.3 Participants

In order to qualify for this study, participant had to 1) be age 65 or older, 2) have a cognitive concern, and 3) use or be interested in using some form of videoconferencing to manage everyday life or engage in enjoyable activities.

In line with critical Disability Studies [37, 51] and the understanding that cognitive concerns exist on a spectrum [6, 39, 69], we recruited without restricting to a particular diagnosis. Our study included people who self-identified as having *cognitive concerns* from the following categories: subjective cognitive decline, mild cognitive impairment, or dementia. This helped us find participants with a range of cognitive concerns, spanning from non-clinical (subjective cognitive decline) to the diagnosed categories of MCI and dementia. The inclusion criteria for subjective cognitive decline were that participants perceived themselves as experiencing confusion or memory issues. Over the course of our research, participants gave more details. For example, participants described their subjective cognitive decline with instances such as, “*I forget things due to grief*,” “*I was just watching something that I cannot remember*,” or difficulty remembering the names of close contacts. While we did not ask for diagnoses, two people said they have Parkinson’s disease, with one also having Lewy body dementia. The remainder described cognitive changes varying from grief to age-related.

We recruited 17 participants. Twelve participants were from independent or assisted living (Community A), four from Center B, and one from Forum C. The average age of participants was 72 years (standard deviation 9.5). Seventeen participants were female and two males. Table 1 provides additional details about participants.

### 3.4 Data Analysis

We employed reflexive thematic analysis to analyze the data [8]. Our data analysis included three phases:

Phase I: The first author familiarized themselves with the data by listening to all audio recordings to verify the transcripts (which had transcribed using Otter.ai [67]), simultaneously creating memos. The first four interviews were then inductively open coded by the first author. During the initial coding process, the first and last author started to become interested in codes that involved participants presenting themselves: “*checking*” and “*mirroring*,” which aligns with behavior typically observed on the “*frontstage*”; other codes such as “*fumbling through*,” “*self-identity*,” and “*role*” seemed to reveal more covert, private aspects akin to backstage activity. We also captured a theme of ‘*adaptation*’ in the realm of videoconferencing including codes like “*transferring to virtual settings*” and “*adapting to changes*”.

Phase II: We inductively grouped initial codes into categories such as tools, physical settings, and background, as well as identities while videoconferencing. We recognized that Goffman’s framework [22] might be a useful lens to understand the behaviors, objects, and identities in the data. After turning to Goffman’s writing and how it has been adopted in HCI, including for disability, we settled on this framework to connect our codes. In particular, we found the notions of frontstage, backstage, and roles as a particularly good fit for our data, and merged existing codes and categories under these concepts

Phase III: We deductively coded all transcripts with the above categories. As we coded the data, some new subcategories emerged, such as backstage. In this deductive coding stage, we focused on analyzing data, including transcripts and observation notes, through this dramaturgical framework [22], which led to our identifying and refining major themes consistent with those observed in the initial batch of interviews. Having multiple kinds of data – remote and in-person interviews, as well as observation sessions – was key for triangulating our findings. For example, we were able to see a participant pause during a cognitive gap with us during the interview, be quiet during an observation session while engaging in bible study, and then learn in a post-observation session interview about how the participant chose to be quiet to avoid others witnessing a cognitive gap. The first author carefully listened to the full audio of the sessions conducted in Mandarin and discussed with the last author to develop final themes. In the process of selecting the best examples/quotations to present the findings, we selectively translated/transcribed some of the Mandarin data.

## 4 Findings

In this paper, we ask how Goffman’s dramaturgical framework can be applied to study videoconferencing technology use by older adults with cognitive concerns. Our findings utilized three sets of theatrical metaphors from Goffman. The first findings section draws on Goffman’s notion of “performances” and “roles,” and particularly the ways participants sustained, adapted, and embraced new roles while experiencing cognitive changes. The second section focuses primarily on “backstage” work, including managing devices and employing “cues”. The third section focuses on the “frontstage”, where participants ensure there is a clear communication channel through which the audience and performer can hear and see each other, and that the “stage,” or participant’s background, is set to communicate the desired impression.

### 4.1 Performances and Roles

Goffman defines a performance as an activity conducted in the presence of, and intended to influence, a set of observers (page 13, [22]). A role is involved in a performance, as “*the enactment of rights and duties attached to a given status*” (page 16, [22]). In the sections that follow, we first provide an overview of the types of performances mentioned by participants. We then describe how videoconferencing was used to sustain, adapt, and embrace new roles in videoconferencing as people managed their cognitive concerns.

#### 4.1.1 Performances.

Participants used videoconferencing to engage in a variety of performance types spanning from more common performance including connecting with their families (P2, P4, P5, P6, P7, P10, P11, P13, P14) and engaging in spiritual and religious practices (P6, P7, P11, P12, P13, P15), to less common performances like engaging in medical videoconferencing discussions with other health professional (P9), managing financial assets with their stock agent (P3), therapy sessions (P8), and dementia support groups (P12). Family interactions were very common, where participants used videoconferencing for routine communications and also more formal get-togethers. The most often described familial performances involved casual conversations and greetings between family members. The goal of these interactions was to stay connected with families, particularly those who lived at a distance. For example, P7 used videoconferencing to regularly communicate with her children all residing in a different country. While casual conversation was most common in familial context, during tech discussion groups, one participant also discussed using videoconferencing for more formal family events, such as participating in anniversary celebration and birthday parties.

A second common type of performance was for spiritual purposes. This allowed participants to extend their religious practices beyond physical venues. The notable characteristics that participants shared about these performances included size and diversity that they would not be able to experience in a physically co-located setting. P12 explained that through the use of videoconferencing, she could participate with “*hundreds of people on these Zoom calls*,” with other Buddhist attendees. Other participants noted that the geographical diversity that videoconferencing supported contributed positively to religious performances. P11 attended a weekly prayer session via Zoom and expressed her excitement about how this prayer session brought individuals from “*different churches*” together from “*everywhere*” in the world.

#### 4.1.2 Roles.

“*Roles*” related to how participants perceived themselves and performed for others. In videoconferencing performance, participants assumed various roles, such as ”*engaged parent*” or ”*devoted worshiper*” that reflected ongoing commitments and values that they held and wished to portray to others. Below, we describe how videoconferencing was used to sustain and adapt participant roles as they experienced cognitive changes, as well as new role opportunities that videoconferencing provided due to their cognitive changes.

##### Sustaining professional roles

Participants used opportunities afforded by videoconferencing to continue engaging with roles associated with their former professional lives even as they experienced cognitive changes. P9 was a trained specialist in internal medicine and geriatrics. Since retiring eight years ago, P9 has remained actively involved in his field, striving to ”*keep up with medicine, especially with whatever current medical problems we’re working with*”. He regularly reads medical journals and participates in several medical videoconferences each month, including for a large national advocacy program and weekly forums at a local university. His videoconferencing performances involve discussions with other health professionals, keeping him connected to ongoing medical debates and working towards his ultimate goal to “*help some people*.” Through these engagements on videoconferencing, P9 has gradually adopted a more advocacy-oriented role. His focus has shifted towards health insurance issues, where he has expressed concerns about the trend towards “*privatizing*” federal health programs.

Even as P9 sustains and deepens his role as a medical professional contributing to society through videoconferencing, he shares how his struggles with the ‘multiple problems of aging,’ particularly cognitive challenges, impact his ability to contribute effectively to his field. He explained that it now “*takes me longer to do something, it takes me longer to understand it*.” The fast pace of speech in videoconferences and unfamiliar terminology can be confusing for him. P9 explained what it felt like to sometimes not know how to do things on videoconferencing as, “*it’s embarrassing, because I’m dealing with a whole bunch of younger crews out there that are running things, and I’m in there, futzing around*.” Although videoconferencing has enabled him to maintain a professional role, perceived generational differences and cognitive decline do affect his ability to perform his role within the professional community.

##### Adapting roles through videoconferencing

For some participants, sustaining their roles can involve modifying the duties associated with them, which they do using affordances of videoconferencing. P3, who was diagnosed with Parkinson’s disease dementia, was able to actively manage her financial investment at her own home through videoconferencing, engaging with financial experts in the stock market. This was coupled with occasional support of her daughter, who helped her to “*set up*” and connect to the stock agent on videoconferences or to explain something from the agent when she didn’t understand. The level of her daughter’s involvement was intentionally managed by P3 as she desired to “*keep some of that to yourself* ”. She described the specifics of her involvement as having “*financial arrangement with stock people*,” enabling her to “*talk*”, “*make decisions*”, “*transact*”, or “*get suggestions*” remotely. P8 found videoconferencing beneficial for therapy sessions, with the affordances of videoconferencing allowing him to “*effectively*” communicate all necessary aspects of the therapy process. P8 favored virtual sessions over traditional in-person meetings post-pandemic. Videoconferencing was not just an interim solution but served as a capable medium to *sustain* the role of a participant in therapy, regardless of physical constraints.

In addition to supporting the alteration of the duties associated with a particular role to accommodate transportation or pandemic associated concerns, videoconferencing supported the adaptation of roles in response to cognitive changes. P8 told us about the “gaps” he experiences in his memory, where it can take him some time to remember something. We were able to see this during the interview in moments where P8 lost his stream of speaking, “*great, my brain…that sucks*…” and then after several seconds continuing, “*fine, my brain came back*” and resuming the thought. However, after we observed P8 taking on a role as a member within a local religious community where he attends regular bible study group through videoconferences, P8 told the researcher he didn’t encounter any cognitive lapse during the videoconference. We can understand how this came about by seeing that P8 took a passive, listening role during bible study, barely speaking during the one-hour videoconference. P8 shared that this was done with intention: he chose to speak less in this group setting to minimize the impact of his cognitive lapses. This strategic alteration in his role demonstrates a conscious effort to remain engaged while managing how he wanted to present his cognitive constraints.

##### Embracing new roles by navigating cognitive changes

In another instance, cognitive changes led to a new kind of role. For P12, diagnosed with dementia, these changes have catalyzed her transformation into a patient advocate, a role that then led to many changes in her life. She moved close to her friends, reactivated her LinkedIn account, shared her diagnosis publicly, and began participating frequently in videoconferences related to dementia, such as the online support group meetings. P12 elaborated on her role as involving “*get[ting] the information out there*” about dementia and supporting other older adults going through memory and other cognitive concerns in their lives. While taking on a new role in the manner described, P12 appears to have been naturally been influenced by her past professional role as a television producer. Describing others with dementia attending support group meetings, P12 said that “*the participants don’t know how to set up even how to be seen visually, sometimes all you see is like a forehead and some eyes, others are in complete darkness. I’ve had to ask them, the TV producer in me ask them - could you try and turn your screen, so there’s more light on you. So, we can… be able to see your nice eyes and listen to you.”*. P12’s instinct was to offer guidance, suggesting adjustments to improve their setup. This inclination to help, rooted in her professional experience, highlights how her past careers can inform and enrich her role in videoconference.

### 4.2 Working Backstage: preparing for the performance

In the ”*backstage*” of performances, individuals engage in more private behaviors, allowing themselves the freedom to relax and be authentic without the audience’s gaze (page 112, [22]). In the findings section above, the backstage came up most clearly when P8 told us about how he had chosen not to speak in the larger bible study session to avoid others witnessing any cognitive gaps: we can see this as P8 and the first author chatting in the backstage more informally about something that had gone on during the performance in the frontstage. We discuss here the role of backstage work in preparing for the frontstage performance. First, participants selected devices that would support a videoconferencing performance appropriately, with relevant characteristics including size, portability, and obsolescence. Second, participants employed tools such as emails and written notes to manage the logistical aspects of preparing for a videoconference.

#### 4.2.1 Selecting Devices for a Smooth Performance.

Device characteristics such as size, portability, and obsolescence are all considered by participants in the backstage. Further, participants maneuvered around physical setups that affected video conferencing activities.

Participants used a range of devices to engage in videoconferences, including smartphones, laptops, desktops, and tablets. Some participants owned multiple devices, but this was never a case of participants having a surplus of advanced devices – often, they navigated shortcomings of different devices or worked with devices that were becoming obsolete.

Participants highlighted the small form factor or portability of the smartphone as pivotal in managing the transition between private (backstage) and public (frontstage) spaces. For instance, P12 multitasked while waiting for her coffee to brew, using her smartphone—which she described as her ”*go-to device*” due to its convenience for checking emails and accessing Zoom meeting links. A smartphone serves as a quick way of accessing frontstage performances, allowing P9 to “*call in*” to videoconferences that enabled him to continue contributing to his field. Yet, there can be a trade-off of quickly transiting between the back and frontstage with compromising other functionality, such as the size of the visible screen on smartphones, that affects the ability to perform certain roles. P9 explained that for some purposes, with a small screen, “*it’s horrible…you can’t read the charts, they are too small*.” As a result, P9 and others such as P11 preferred laptops for videoconferences where they benefited from a larger display and enhanced functionality.

Participants deliberately organize their technologies in their homes in face of power availability and device size to support their videoconferencing practices, which is a form of backstage preparation for frontstage performances. For instance, P14 set up a designated table with her tablet on it next to a power outlet, to keep the device’s power sustained through long videoconferencing sessions. Multiple participants, including P8 and P3, had centralized electronic zones with computers alongside their TVs. P3’s computer setup in the living room next to the couch and TV was designed to enhance comfort, facilitating frequent email checks and logistical planning as backstage preparations before video conferences throughout the day. This physical organization of devices can sometimes hinder performance in videoconferences or home life. P11 recounted a scenario where her videoconferencing was delayed because her laptop on the living room desk couldn’t be easily moved to troubleshoot technical issues. P9’s laptop was placed on the dining table where he and his wife have meals every day, which he described as “*unfortunate*” but necessary due to “*space constraints*” in his home.

Finally, device obsolescence affected the work done in the backstage to prepare for frontstage performances. Participants had devices that were years or even over a decade old. When older technologies no longer supported efficient videoconferencing, participants choose different devices (with different affordances impacting performance, as described above). P1 shared how the outdated nature of one of her devices, given to her by her family, prompted her to abandon it for videoconferencing: “*Sometimes I’ll set up my computer. I don’t use my iPad much anymore because it’s getting older*.” Devices becoming obsolete can add to the backstage workload for participants. P9 had a daily routine before going to bed:

“I have not replaced my computer since I retired… It had barely any memory to begin with in those days… I have to run the cleaner [program] every night before I go to bed so there is space for me go on and get it going in the morning.”

Even this backstage work does not guarantee the ability to engage in a videoconferencing performance. P9 explained “*It’s always stressful till you’re on [the call]*”, describing the uncertainty involved in managing their old, slow computer: “*You wait for a while, and you decide it’s not doing anything. Part of the time it was still working and part of the time, it’s not doing anything… And then sometimes it’s insolvable. You don’t ever get on the meeting*.”

#### 4.2.2 Logistics work through the use of “cues”: emails, handwritten notes.

One participant in the tech discussion group explained how videoconferencing is “a lot easier than going someplace [physically] and waiting for somebody to encounter with all the delays and stuff that happened.” This observation is consistent with our findings that videoconferencing, unlike in-person gatherings where performances can occur spontaneously, generally adheres to a structured schedule. This included pre-scheduled church sessions, bible studies, and family meetings. This meant, however, that to engage in performance on time, particularly in face of cognitive concerns, participants needed to conduct substantial logistical work in the backstage.

Participants conducted logistical work utilizing, in Goffman’s terms, “*cues, hints, and stage direction*” to prepare to begin performances at the right time, on the right day (page 72, [22]). Our analysis reveals the importance of digital cues, like emails created by others, as well as physical cues, such as handwritten notes created by participants themselves.

Emails frequently serve as such ”cues” which guided participants into meetings. As P3 said, the emails “*pass a message*,” or more specifically, “come out with a number [videoconference link]”. By clicking this digital link in emails, participants are directed to the videoconference “stage” to perform. It seemed that these kinds of cues were particularly important given cognitive changes. P12 described her experience preparing for a videoconference interview with the researcher as “sheer hell”. She had explained that using videoconferencing has become “*very, very difficult*” and expressed gratitude for the reminder email sent a day before the meeting so she could remember the meeting.

”Cues” are not limited to digital reminders sent by others like email; they also encompass physical elements created by participants in their daily life. Handwritten notes were used by many participants such as P8, P12, P11, and P14 to manage logistics. For instance, P12 marked the dates and times of videoconferences on her whiteboard to maintain awareness of them. Similar to the digital cue as email, handwritten notes facilitated a smoother videoconferencing performance. During the interview with P11, as the researcher demonstrated a feature of videoconferencing on his screen, P11 said, “*I should be taking notes now*,” because she thought our conversation about videoconferencing was informative. She describes this process of taking handwritten notes as a strategy employed often in her everyday life, done here to create a ”*double impression*” in her mind that could aid the retention of information. Another participant, acknowledging her cognitive decline, anticipated adopting notetaking soon, as she found it increasingly difficult to concentrate on specific details: “*It is hard to pay attention to some certain things*”. We can view the utilization of handwritten notes as a borrowed practice from participants’ daily lives to reinforce and support their memory.

However, handwritten notes were not always reliable as cues to ensure a timely performance. One reason was difficulty maintaining awareness of physical notes. P12 shared how she forgets to look at where she noted dates of a Zoom meeting, which led to missing the meeting. Readability is another challenge. P9 faced challenges in reading her own writing, which she described as ”scribbly”: “*So, you write something down to remember it, and you remember it when you look at it but you can’t decipher seeing it*”. While notes are intended to aid cognition, their utility is compromised if they are not legible or easily decipherable. P9’s experience underlines the importance of ensuring that such physical cues are not just created, but are also practical and accessible.

### 4.3 Self-presentation through stage management

In Sec 4.1, we explored how participants use videoconferencing for various performances, as well as how videoconferencing was used to sustain, adapt, and embrace new roles as they managed cognitive concerns. Then, in Sec 4.2, we described often-invisible backstage efforts, highlighting how participants select devices based on specific characteristics and utilize tools to support logistical preparations before their frontstage performances. This section of our findings moves to the frontstage, revealing how participants manage their impressions through videoconferencing.

The frontstage is “*where the performance is given*” (page 107, [22]). Participants found the audio and video channels afforded by videoconferences as important for giving performances and communicating with audiences. To make these communication channels work for their performances, participants engaged in two kinds of work. First, they employed sounds and visual checks to ensure a smooth connection. Second, they set backgrounds to convey certain impressions.

#### 4.3.1 Sound and Video Check: Obtaining a Clear Communication Avenue.

In ideal cases, as P8 explained regarding their counseling calls, a videoconference affords a clear avenue for communication: “*We see each other, we hear each other, and everything that needs to be communicated can be effectively communicated*”. However, many participants described cases to us where their communication channels were not working, disrupting a performance. Several participants raised concerns about properly presenting their image and speech from past videoconferencing experiences. P11 said that sometimes during videoconferencing calls with her family, “*I could see them, they couldn’t see me*.” When P12 tried to meet her long-distance grandchild through videoconference, she was informed that “*Nana (grandmother), we can see you, but we cannot hear you*.”

Participants demonstrated an understanding of the features that provided basic control of audio and video such as the “mute” and “show video” buttons. To have a clear communication channel, however, it was not enough to know how to operate the controls that were built into their videoconferencing platform. Participants had to set up their space in advance. P3 described preparing for visibility for her financial videoconferencing meeting by arranging the physical setting to accommodate multiple participants within the range of her camera. She noted, “*… the three of us will just pull up chairs like you do, and formation that ought to be seen*.” P8 tilted and adjusted his camera and setup to make sure what he wanted to capture was being captured before he logged onto his bible study call.

Beyond setup, participants had to engage in a continual focus on checking and adjusting these channels to deliver their performance. This occurred during the interviews that we conducted over videoconferencing. We noted that it was not always straightforward to determine if video or audio was working. Approximately one minute into the conversation, P12 apologized for not turning her video on, “*Right. Oh, I’m sorry. I don’t have my video started. There you go*.” Reflecting on why he had not asked them to turn on their video immediately when the interview started, the researcher noted that he was not sure whether the participant had wanted their camera on. This experience revealed a social norm governing our own expectations around videoconferencing, but also the importance of a performer being able to monitor what the audience can see by looking at the image of themselves on Zoom. Sometimes, participants would use this image of themselves but still check if they were appearing well to their audience: during the beginning of a videoconferencing call, P8 offered to adjust his background for the researcher. P8 said, “*I can turn on the lighting and get some background lighting, is that necessary?*”. During the same call, the researcher asked several questions to the participants and received unclear responses like “I’m sorry?”, “Am I…?”, “No…”, it became evident that P8 was having difficulty hearing. The researcher then checked, ”*Can you hear me? Is my volume alright?*” confirming P8’s auditory challenge as he replied he could ”just barely” hear. Reacting quickly, P8 adjusted his camera to display another aspect of his background to the researcher, attempting to continue the conversation visually. Although the researcher made no adjustments, P8 continued to hear and respond without further issues for the remainder of the session. P8 later revealed that: “The volume on the laptop is not quite sufficient for me to hear you (the researcher) clearly.” His strategy was to put on headphones, “Because the headphones amplify the volume to a point where I can hear you, it’s extremely useful.” P8 may have additional “backstage strategies” – his use of captions was not observed in this study, but P8 described captions as a “secondary device” that helps him to “communicate” in cases where he can’t hear but the “caption at least approximates the meaning of the words.”

#### 4.3.2 Setting the Stage: Managing Impressions through the Background.

Goffman describes the setting of performance to include “furniture, décor, physical lay-out, and other background items which supply the scenery and stage props for the spate of human action played out before, within, or upon it” (page 13, [22]). Individuals use these elements of their physical environment to convey a desired impression to their audience [22]. In our data, this was most evident through the ways that participants presented particular background scenery to their audience through videoconferencing, just like actors use different backdrops to cater to a specific performance on stage.

Participants tailored their physical space to craft particular backdrops to create specific impressions for their audiences during frontstage performances. P8’s setup with his computer and TV on the same table capture a bookshelf as the background while ensuring adequate lighting. While P8 did not explain the impression they intended to convey with their bookshelf in the background, it connotes a scholarly impression which matches the way the participant later described an interest in learning about a wide variety of topics (“*I don’t watch TV, but let’s go into YouTube, on various YouTube channels on various topics from religion, arts and crafts, history, and whatnot*…”). While they could not attain it at the present time, P15 expressed a desire for a virtual background that resonates with the theme of religious videoconferences to enhance the sense of spirituality in these gatherings. Not all impression management was for formal purposes: P14 uses videoconferencing to maintain a personal connection with her brother, who lives in a country with a 12-hour time difference. P14 and her bother chose a particular time when P14 was going to sleep and her brother just woke up from bed, with both of them presenting their headboards in the background. This particular background was “fun” in a nostalgic sense described by P14, “*Our parents are gone, and now this [headboard in the background] is really fun! It is like we two are going back to childhood*.” These choices reflect an awareness of participants for how they wanted to present themselves in the virtual environment, highlighting the role of managing physical space for digital impression management.

The importance of being able to use a background, including virtual backgrounds, to present a desired impression is highlighted in cases where participants describe not being able to do so (P11, P12, P16, P17). One participant described being unable to adjust her background to present the impression that she wanted, leading her to opt out of Zoom meetings: *“…I belong to a lot of different organizations that require the use of zoom for things or to participate in meetings. And a lot of times, I’ll just opt out, because, yeah, I can’t figure out the backgrounds, like how to do the backgrounds, and I’ll have some weird thing with Christmas lights over me on like, what is going on? You know, and I don’t know how to get rid of it. Until after the fact. And, you know, just again, those are those icons that aren’t, you have to go to many layers down to find how to get in and out of the settings*.” Adding or changing virtual backgrounds seemed to be an unknown area for another participant as well, who said “*okay, that’s another thing I’d love to learn how to make that*” when the researcher demonstrated that background could be adjusted to a virtual background [P11].

Not all elements of the background can be controlled in advance, requiring performers to improvise to manage the audience’s impression. During a Zoom interview with P12, their cat made an appearance three times. The first instance occurred while P12 was discussing her online dementia group. Upon noticing the researcher’s awareness of a sudden “*meow*,” P12 apologized for the background noise. Subsequently, she explained the situation with her cat, stating, ”*My cat is sitting in her kibble bowl, and she had a physical tic*.” In response, the researcher mentioned, ”*My cat is right next to me as well!” Encouraged by this, P12 shared more details about her cat’s behavior,* ” Literally, she bangs her head, throws it in the air, and then eats it. But she’s as happy as a clam.” Following this, she smoothly redirected the conversation back to the original topic with an ”anyway.” The cat made its second appearance during the conversation when P12 mentioned that the cat “just fell off the window” in her background. This exchange led to further conversation about the cat between the researcher and P12, including the cat’s age and how P12 came to adopt it. The final appearance occurred near the end of the videoconference, with P12 saying that she needed to take care of her “*crazy cat*” after expressing gratitude to the researcher, signaling the end of their chat. When a background can’t be controlled in advance, people can use strategies to manage others’ impressions of them – in this case, with P12 as a bemused but fond owner of a slightly bizarre cat.

## 5 DISCUSSION

Our findings answered our research question on how Goffman’s dramaturgical framework [22] can be applied to study videoconferencing technology use by older adults with cognitive concerns by deconstructing our data into three sets of theatrical metaphors: roles and performances, the backstage, and the frontstage. Below, we discuss insights, design implications, and future areas for exploration that come from this work.

### 5.1 Meaningful Engagement through Videoconferencing

Videoconferencing technology is often discussed as a way to deliver services to older adults [30, 41], and specifically for older adults with cognitive concerns (e.g., health services [24, 27, 34], social support [25]. Viewing the data through Goffman’s theatrical metaphors positions people with cognitive concerns as the “main character” in these interactions, which allows us to see the active role they are taking in selecting videoconferencing opportunities that are relevant to them, using the affordances of videoconferencing, and selecting the roles they wish to play.

We have observed more personal uses of videoconferencing technology than found in past work. Previous research has primarily focused on videoconferencing within collective social behaviors, such as cognitive assessments [24, 27, 34] and facilitating social connections through online groups [25] to support and assist cognitive concerns, rather than individual interests. We found videoconferencing used to engage for individualized purposes such as therapy sessions, religious gatherings, financial management, continuing education, and contributing back to society. While individuals with cognitive concerns may certainly benefit from use specific to cognitive concerns as studied in past work, they are also using and benefiting from the same kinds of remote interactions as others. The range of use in our data aligns with Zhao et al.’s taxonomy of meaningful online participation for older adults, which included four levels: communication, collaboration, lifelong learning, and societal contribution [64]. We also found one additional level, which was spiritual engagement. Our discovery of these performance types indicates that some older adults with cognitive concerns are using videoconferencing to engage in meaningful online participation. Their usage can reveal the interests that they see as relevant to their lives.

Past work has sought to understand how to create accessible videoconferencing engagements in the context of disability [31, 32, 72]. For example, a videoconferencing platform was specifically developed to facilitate communication for people with hearing disabilities in mixed hearing groups [72]. Their study found different kinds of work and practices participants engaged in adapt to the communication context. Similarly, our work identified forms of work that people with cognitive concerns employed to cope with changes they were experiencing in videoconferences, like use of notes or avoiding speaking in videoconference. However, for many older adults with cognitive concerns, ranging from memory confusions to MCI and dementia, they demonstrate different changes in their lives in this study, leaving uncertainty to find effective accessible videoconferencing. For example, they notice initial memory confusion, like forgetting friends’ names (P8), or they may have mobility difficulties in their everyday lives due to dementia (P3). Our work adds an additional perspective for future accessibility studies on videoconferencing by identifying some work and practices that may not directly caused by their cognitive concerns, such as sound and video check to obtain a clear communication avenue, or preparing for setting the stage to manage the impressions. This work showed that although the participants had to manage cognitive concerns, they also engaged in other kinds of work necessary for videoconferencing. Shifting away from deficit framing [61] (i.e., not framing cognitive concerns as “problem” that can be managed by technology), our findings around roles (Sec 4.1) suggests a continuation of life-long perspectives through the use of videoconferencing: to sustain professional roles, make changes to existing roles, or find new roles. Beyond creating videoconferencing designs that caters to people’s cognitive disabilities, there also appears to be space for us to study the work people with cognitive concerns engage in to help them continue their roles and values in videoconferencing.

As discussed above, there is space for new and novel approaches that explore the in-depth connection between the roles people perceive in videoconferencing based on their life-long experience (e.g., dementia advocate) and how they want videoconferencing to help them present those roles (e.g., being able to see and talk to other people living with dementia through videoconferencing). To build this connection, it requires a more thorough understanding of both people’s everyday lives and their technology uses. To embrace this idea, we can incorporate perspectives that support a higher-level view of existing use such as technology habituation [2, 54], or ecosystems [10, 17].

### 5.2 Becoming Aware of Backstage Towards More Accessible Videoconferencing for Older Users

Previous work in HCI has drawn on Goffman’s work to understand the kinds of behaviors people engage in private (the backstage) versus public (the frontstage) in the mediated communication technology settings [3, 21, 35, 48, 74, 75]. Past work by Aoki et al. reveals the importance of backstage work for a smoothly functioning performance [3]. In their military context, the backstage is a confidential area where activities are done to support the execution of commands in the frontstage. One example is the creation of encrypted messages, or “voice nets,” that are broadcasted to the frontstage for use by command personnel. Our work also uncovered some specific mechanisms through which backstage messages support frontstage performances. First, others can send emails with videoconference times and links to participants (which we referred to as “cues”). Participants then check (and re-check) emails for information and updates, and use additional mechanisms to capture this information and surface it in a way that will be accessible. An additional key component of backstage work was choosing appropriate devices by considering different aspects, including setting up devices next to a power outlet or choosing a larger device for better visuals during a videoconference.

Moving forward, researchers and practitioners can continue to explore the forms of work necessary for smooth videoconferencing performances, and how to support people with cognitive concerns and others in their support networks in doing this work. We believe that working on this topic in parallel with other CSCW work studying complex communication situations offers another way to move away from a deficit framing, which is easy to slip into in work with older people [61] and people with cognitive concerns [29]. Instead of seeing participants such as those in our study as doing something wrong when technology does not work well (e.g., audio not working during a call), we can understand the importance of factors outside of the individual. For example, we can turn our attention to how devices that participants have access to can be very old and require daily care. Overall, there is a lot of complexity in what it takes to participate in videoconferencing.

One example of a non-deficit lens on videoconferencing that could be employed usefully for future work based on our findings is studying the temporal coordination required across the back and frontstage for videoconferencing engagements. The framing of temporal coordination had been employed usefully in HCI in different contexts including ride-sharing services mediated by algorithms [45], spatially dispersed teams using augmented reality-based assistance systems [57], and medical settings with complex coordination and scheduling workflows [5, 46]. Bardram explores the notion of temporal coordination on three different levels: 1) how it is achieved, 2) how it is managed, and 3) how it is shaped [5].

We further our discussion drawing insight from each of Bardram’s levels. First, temporal coordination is an activity which seeks to integrate distributed collaborative actions [5]. In our case, this collaborative action is between the backstage and frontstage (e.g., the emails and notes described in discussion with Aoki et al. ‘s work [3] above). However, even when backstage supported participants in “getting to” the frontstage, participants still faced uncertainty about whether their audio and visual channels worked – as these could only be checked during the videoconferencing performance itself. In this case, researchers can support certain tasks in the backstage instead of the frontstage to enable better management of frontstage impressions. That means that instead of leaving people uncertain about the status of their audio and video when they interact with others, there could be prompts made while participants prepare for a videoconference to help them verify audio and video.

Second, in elaborating on how temporal coordination should be managed, Bardram provides the best practice of “establishing starting time and deadlines” [5]. The specific example is of a clock in a clinic representing the “official time,” according to which all activities are recorded and synchronized. Similarly, we found logistical tools people used to manage the temporal coordination including “cues.” However, the use of those tools as we found were not stable, for example in the case of “scribbly” handwritten notes or forgetting to use the whiteboard. Notably, there will be a need to better structure the coordination tool to connect and streamline the work people engage in both backstage and frontstage. There are opportunities for videoconferencing software to better structure backstage practices, such as digitizing distributed physical notes across all stages so they can be better accessed and shared whenever needed by users.

Lastly, Bardram emphasized that the practical process of temporal coordination cannot be detached from conditions of the concrete situation. Specifically for this level, we are in need of future work that considers how engaged partners like caregivers are involved in temporal coordination. While our work did not focus on caregivers or the people sending videoconferencing links for access, there were occasions where other engaged partners appear in participant stories. For instance, P3’s daughter helped her videoconference at times, and P1 had a device gifted by a family member. This aligns with past research, which reveals that others play important roles, particularly for participants with more advanced cognitive concerns, and should be incorporated into research as well [76].

### 5.3 The Importance of Impression Management

Self-presentation came up in the ways that people managed decisions about whether and how to show their cognitive concerns as well as the ways that they managed audio, video, and their physical setting (backgrounds) to ensure that their audiences could see and hear them as they wished to be seen and heard.

Researchers should examine the impact of presenting cognitive concerns in mediated communication technology such as videoconferencing, as this appears to affect the experience of people using them. P9 described feeling embarrassment when their cognitive concerns slowed down their responses to fast-paced speech and unfamiliar terminology during interaction with younger audiences during videoconferencing with their professional medical community. Our findings reveal that participants present their cognitive concerns differently from each other in the context of videoconferencing. This is consistent with Zhu et al.’s discussion of disability disclosure, which notes that individuals adopt varied practices to present their disabilities [65]. Different disclosure practices corresponded with different challenges in Zhu et al.’s work [65] as well as ours. When P8 spoke less to make his cognitive lapse less visible, it resulted in lowered participation. P12 openly disclosed her cognitive concerns to the researcher with apologies for how it led to her confusion about operating videoconferencing settings. A challenge with this kind of disclosure is the stigma that can emerge due to audience biases when disability is discussed openly [43, 65]. For people with cognitive concerns, a specific risk may be whether the audience sees their accounts as trustworthy or reliable after hearing their disclosure. To alleviate the challenges of stigmatization and audience biases faced by people with cognitive disabilities, we propose an addition to existing frontstage work (e.g., sound and video check) by integrating an optional cognitive load detector through eye-tracking technology during a videoconference call. This aims to help individuals be aware of their cognitive status and take action if when they want to during the call. This design approach can be further extended to provide people with the option to share their cognitive load with others on videoconference, avoiding the need to verbally disclose any cognitive status directly.

Our findings also remind us that there are impressions that people with cognitive concerns seek to manage beyond their cognitive status. Backgrounds are one affordance of videoconferencing that supports participants in conveying certain impressions. Participants tailored their physical space to craft a particular backdrop for a specific impression. For instance, P14 created a background of his bed frame to match his brother, who is hard to meet, which led to reminiscing about childhood memories. This is similar to Roulin et al. ‘s work, where people composed their surroundings, like leaving mugs with the same symbols visible to show a shared political orientation [49]. P8, who enjoyed pursuing spiritual engagement in bible study, arranged their camera in view of their physical background which included a bookshelf. His efforts in self-presentation were likely rewarded: prior literature studying how videoconferencing background affect perceptions has found that bookcase background was consistently rated as most trustworthy and competent [13]. Virtual backgrounds provide one way to manage impressions, such as P15 desiring a background with religious symbols. However, participants expressed not knowing how to use virtual background. With different roles and performances people take in videoconferencing, future work can support people in conveying desired impressions by focusing on specific affordances such as the virtual background.

## 6 Conclusion

Current approaches to study videoconferencing technologies share our goal of exploring its potential and making the technology more accessible for people with diverse abilities. A lens we adopted to study videoconferencing with people with cognitive concerns is a dramaturgical framework by Ervin Goffman. We studied the “roles” people play during the performances, their “backstage” work entangled in a physical world that they adapt to videoconferencing, and the “frontstage” impressions that they manage to communicate with their audiences. Through further discussion with existing literature, we expand on each of these dimensions: on designing videoconferencing technology for meaningful roles, for adopting CSCW framings to further unpack and support the videoconferencing ecosystem, and for supporting impression management in light of what is known about disability disclosure with other groups.

## 7 Limitations

Our study has several limitations. This includes the possibility of selection bias due to our convenience sampling method. We employed convenience sampling broaden our participant pool, addressing the challenges of recruiting individuals with cognitive concerns as highlighted in past research (e.g., [16]). It is important for future research to continue to explore best practices for recruiting this demographic for technology research.

## Figures and Tables

**Table 1:** Participant Demographics.

PiD	Age Range	Method of Data Collection	Race	Cognitive Concern
P1	60–69	Discussion, interview	White	Subjective cognitive decline (grief-related)
P2	80–89	Interview	White	Diagnosed with dementia
P3	90–99	Interview, observation	White	Diagnosed with dementia
P4	80–89	Discussion	White	Subjective cognitive decline
P5	90–99	Discussion	White	Subjective cognitive decline
P6	80–89	Discussion	White	Subjective cognitive decline
P7	90–99	Discussion	Black	Subjective cognitive decline
P8	80–89	Discussion, interview, observation	White	Subjective cognitive decline (age-related)
P9	Unknown	Discussion, interview	White	Subjective cognitive decline (age-related)
P10	70–79	Discussion	White	Subjective cognitive decline
P11	80–89	Discussion, interview	White	Subjective cognitive decline (age-related)
P12	70–79	Discussion, interview, observation	White	Diagnosed with dementia (Lewy Body)
P13	70–79	Discussion	White	Subjective cognitive decline
P14	70–79	Discussion, interview	Asian	Subjective cognitive decline (age-related)
P15	80–89	Discussion, interview, observation	Asian	Subjective cognitive decline (age-related)
P16	70–79	Discussion, interview	Asian	Subjective cognitive decline (age-related)
P17	80–89	Discussion, observation	Asian	Subjective cognitive decline (age-related)

## CCS Concepts

• Human-centered computing; • Human computer interaction (HCI); Social and professional topics; • User characteristics; • Age; People with disabilities;
